# A broader view on outpatient parenteral antibiotic therapy (OPAT) stewardship

**DOI:** 10.1017/ash.2021.252

**Published:** 2022-01-31

**Authors:** Danielle Hess, Christina G. Rivera, Monica V. Mahoney

**Affiliations:** 1 Department of Pharmacy, Mayo Clinic, Rochester, Minnesota; 2 Department of Pharmacy, Beth Israel Deaconess Medical Center, Boston, Masschusetts

*To the Editor—*We read with interest the recent publication by Brenon et al^1^ regarding the overuse of broad-spectrum outpatient parenteral antibiotic therapy (OPAT). As noted by the authors, broad-spectrum antibiotics with once-daily dosing are often chosen for OPAT due to ease of administration despite options with a narrower-spectrum that may require multiple administrations daily. We applaud the authors for highlighting stewardship opportunities in OPAT, which is an underexplored field within antimicrobial stewardship (AMS) practice and research. Most intravenous (IV) antibiotic courses are completed in the outpatient setting, and AMS through OPAT programs is a significant and underserved need.

While spectrum of activity based on culture and susceptibilities is key in determining appropriate antibiotic therapy, considering OPAT regimen selection with a broader stewardship lens is likewise essential.^2^ Patient-specific factors, such as indication, allergies, organ function, previous drug intolerances, cost, risk of adverse events, and drug–drug interactions, play a large role. In addition to these factors, discharge location, ease of administration, caregiver or patient time and effort, and drug stability must also be considered before concluding that an OPAT regimen is a suboptimal choice.

Within the purview of AMS, one must consider whether the given regimen will be compatible with the patient’s life or whether the complexity of administration could lead to nonadherence. Hamad et al^3^ conducted a survey to determine the rate of nonadherence with OPAT regimens and associated factors leading to nonadherence. The survey results revealed that 10% of patients were nonadherent due to younger age, household income <$20,000, or lack of time for administering IV antibiotics. Conversely, less frequent dosing and having the support of a friend or family member during IV antibiotic administration were associated with better adherence.^3^ Considering further how ease of administration affects adherence, a meta-analysis comparing compliance with once-, twice-, or thrice-daily administration showed that lower frequency dosing led to higher compliance rates regardless of the study design or treatment duration across 26 randomized controlled studies.^4^ Thus, it is essential to consider patient adherence along with the spectrum of activity, especially if “narrowing the spectrum” requires a patient to complete multiple IV antibiotic administrations daily. Hospital readmission and/or infection recurrence arising from nonadherence would incur significant costs and could result in compounding antimicrobial usage. Although converting standard bolus antimicrobials to continuous infusions via a continuous ambulatory delivery device (CADD) might appear to be a ready solution, certain antimicrobials (including ampicillin) cannot readily be given using this method due to drug stability. Also, insurance companies may deny CADD pump coverage. Furthermore, the potential for a patient to need placement in a skilled facility for an OPAT regimen to be administered (due to its frequency) warrants serious consideration in the COVID-19 pandemic era.^5^

Although the threat of antimicrobial resistance is a major incentive to narrow antibiotic therapy, evidence regarding whether more narrow therapy leads to less drug resistance is controversial. A retrospective cohort study by Tartof et al^6^ investigated whether inpatient antibiotic stewardship programs (ASPs) reduced infection rates of high-profile drug-resistant organisms. A key program component was restricting use of broad-spectrum antimicrobials, including ceftriaxone. With >765,000 hospitalization episodes included, these researchers detected an overall increase in vancomycin-resistant enterococcal infections after this intervention, and they detected no changes in the rates of extended-spectrum β-lactamase, carbapenem-resistant *Enterobacteriaceae*, or multidrug-resistant *Pseudomonas aeruginosa* infections after the intervention. Thus, Tartof et al concluded that ASPs with successful reductions in consumption of targeted antibiotics may not yield changes in antimicrobial resistance patterns in the 2 to 6 years after implementation.^6^

While the impact of ease of administration on a patient’s life is tangible, the definition of a “broad-spectrum” or “broader-spectrum” antimicrobial is largely a subjective, conceptual matter. Various scoring systems have been developed to define antimicrobial spectrum usage. The antibiotic spectrum index (ASI) developed by Gerber et al^7^ classifies antibiotics based on activity against important pathogens. Similarly, a study by Peryrani et al^8^ used the antibiotic intensity score (AIS) calculated as the sum of the number of days of each antibiotic multiplied by the antibiotic spectrum. In their study, Brenon et al^1^ extrapolated National Healthcare Safety Network (NHSN) antibiotic use and resistance antimicrobial groupings, classifying agents as class 1 (broad-spectrum) to class 4 (narrow-spectrum). Notably, the adapted NHSN scoring method was not subject to validation. To illustrate the limitations in applying the score, ceftriaxone was deemed to be a “broader-spectrum” agent for gram-positive infections, but for certain gram-negative infections it may be carbapenem (ertapenem) sparing and thus a “narrowing” agent. These scenarios were not equally considered in the scoring system.

To consider a potential future direction, another AMS approach for OPAT includes IV-to-oral switch therapy (IVOST), which involves safely transitioning patients from IV to oral antibiotic regimens at the optimal time due to clinical improvement and meeting evidence-based and complex outpatient antimicrobial therapy (COpAT) criteria for serious infections. High-quality evidence for IVOST and COpAT is rapidly emerging and may hold the opportunity to avoid the disadvantages of OPAT: using intravenous access and its related complications, weekly or more frequent laboratory monitoring, and home healthcare or infusion center or facility admission requirements.^9^

We applaud Brenon et al for bringing attention to the AMS potential in OPAT. It is crucial to derive AMS interventions that are tailored to the broader OPAT context while allowing the patient to successfully complete their OPAT course, treat their infection, and not use additional healthcare resources.
